# The Fate of Antibiotic Resistance Genes and Their Influential Factors During Large-Scale Cattle Manure Composting

**DOI:** 10.3390/toxics14050428

**Published:** 2026-05-13

**Authors:** Zhuo Sun, Siyu Yang, Tong Zhang, Hongyin Li, Peng Gao, Liqiu Zhang, Li Feng, Qi Han

**Affiliations:** 1 Hebei Key Laboratory for Emerging Contaminants Control and Risk Management, College of Environmental Science and Engineering, Beijing Forestry University, No. 35 East Qinghua Road, Haidian District, Beijing 100083, China; 2 Beijing Key Lab for Source Control Technology of Water Pollution, College of Environmental Science and Engineering, Beijing Forestry University, No. 35 East Qinghua Road, Haidian District, Beijing 100083, China; 3 Engineering Research Center for Water Pollution Source Control & Eco-Remediation, College of Environmental Science and Engineering, Beijing Forestry University, No. 35 East Qinghua Road, Haidian District, Beijing 100083, China

**Keywords:** cattle manure, aerobic composting, antibiotics, antibiotic resistance genes, bacterial community

## Abstract

Animal manure represents a critical reservoir that facilitates the dissemination of antibiotic resistance genes (ARGs) and mobile genetic elements (MGEs). However, the current understanding of ARG evolution during extensive composting remains insufficient. This study systematically investigated two common aerobic composting techniques: push-flow trough composting (FC) and membrane-covered composting (FM). Results indicated that both processes demonstrated substantial antibiotic removal capacities, achieving total removal rates of 88.89% (FC) and 79.20% (FM). Nevertheless, their effectiveness in removing ARGs varied considerably. During the 31 days of composting, the total removal rates of ARGs were 59.97% (FC) and 76.11% (FM), while the removal rates for class 1 integron (*intI1*) were 2.31% (FC) and 69.13% (FM). With the exception of *tetX*, *tetG*, and *tetW*, all other ARGs exhibited a rebound during the later stage of the FC process. In contrast, the FM process effectively reduced the risk of ARG rebound during this phase, which can be attributed to its extended thermophilic period and the physical barrier effect of the semi-permeable membrane. Network analysis indicated that ARGs were primarily associated with *Bacillota* and *Pseudomonadota*. The Partial Least Squares Path Model (PLS-PM) revealed that the bacterial community was the main factor influencing ARG dynamics in FC, while in FM, both the bacterial community and *intI1* were the primary drivers. This study provides critical insights for optimizing composting strategies to prevent the dissemination of antibiotic resistance.

## 1. Introduction

Antibiotics are widely used in animal husbandry for disease prevention and growth promotion [1]. In China, the animal sector accounts for 52% of the total national antibiotic consumption [2]. However, the systemic absorption and metabolism of antibiotics in animals remain remarkably low, with over 70% being excreted via feces and urine as either parent compounds or active metabolites [3]. These substantial antibiotic residues exert strong selective pressure on environmental bacteria, thereby facilitating the emergence and widespread dissemination of ARGs [4,5].

ARGs are distinguished by their heritability, capacity for horizontal gene transfer, environmental persistence, and multifaceted ecological risks [6,7]. Consequently, they pose a more persistent and severe ecological threat than antibiotics themselves. It has been projected that by 2050, nearly 10 million people worldwide will be affected by antibiotic resistance annually, with cumulative healthcare expenses and productivity losses estimated at around 100 trillion US dollars (USD) [8,9]. Animal manure serves as a critical environmental reservoir that promotes the accumulation and dissemination of ARGs [10,11]. The annual production of livestock manure in China has been reported to reach 3.8 billion tons [12], making it a major contributor to agricultural non-point-source pollution [13]. Land application of untreated manure can facilitate the infiltration of ARGs into the soil-crop continuum, ultimately endangering human health via the food chain [14]. Hence, gaining a sophisticated understanding of the evolutionary fate of these contaminants during manure stabilization processes is of critical importance.

Aerobic composting is a cost-effective and widely adopted technology for livestock manure management [15]. This process not only stabilizes organic matter and inactivates pathogens, but also facilitates the removal of organic contaminants. Various studies have demonstrated its efficacy in reducing the levels of most antibiotics [16], although its effectiveness in eliminating ARGs varies. Zhang et al. (2023) [17] reported that approximately 50% of ARGs were eliminated following composting. In contrast, Sun et al. (2024) [18] indicated that over 90% of ARGs were removed during hyperthermophilic composting utilizing a semi-permeable membrane cover. Additionally, Wang et al. (2022) [11] observed that tetracycline and vancomycin resistance genes were enriched during composting.

Push-flow trough composting is a widely adopted technology in large-scale composting facilities. Compared to traditional static trench composting, this method significantly reduces the overall fermentation time. During the turning operation, the materials are thoroughly mixed, moisture is effectively evaporated, and oxygen interacts fully with the materials. However, this composting approach has inherent limitations. Frequent mechanical turning may cause disturbances within the pile and may also lead to potential air pollution challenges, such as dust and odor emissions. In contrast, membrane-covered composting represents an emerging and enhanced treatment technology [19]. This technique employs a semi-permeable membrane, such as expanded polytetrafluoroethylene (e-PTFE), to cover the compost pile, while blowers supply oxygen [20,21]. This composting method effectively mitigates the emission of dust, bioaerosols, and even noxious gases. Nevertheless, this technology also has certain drawbacks. For instance, condensate reflux can easily promote mold growth, and localized heterogeneity in moisture and oxygen levels may occur [22]. Most previous studies have been conducted at a laboratory or pilot scale, where factors such as temperature, oxygen, and moisture can be readily controlled and uniformly distributed. However, when composting is scaled up to practical engineering applications, the conditions become significantly more complex. The substantial physicochemical heterogeneity of materials, limitations in mass and heat transfer, and increased susceptibility to environmental perturbations collectively influence the fate of ARGs. Consequently, the dynamic behavior and driving factors of ARGs during large-scale composting have become an urgent research priority.

Push-flow trough composting and membrane-covered composting are both widely adopted technologies in large-scale composting facilities. Therefore, these two processes were selected as the focus of this study. Samples were collected at various stages of composting to analyze environmental factors, representative antibiotics, ARGs, and MGEs. The specific objectives of this study were: (1) to assess the variation and removal efficiencies of antibiotics, eleven ARGs, and *intI1*; (2) to explore the co-occurrence relationships among ARGs, *intI1*, and bacterial communities; and (3) to characterize the correlations among ARGs, bacterial communities, antibiotics, and environmental factors, while identifying the driving factors behind ARG transmission, thereby providing new insights into the dissemination and control of ARGs during large-scale composting.

## 2. Materials and Methods

### 2.1. Aerobic Composting and Sample Collection

Two representative large-scale composting processes were monitored: push-flow trough composting (FC) and membrane-covered composting (FM). Samples of the FC group were obtained from a facility in Miyun, Beijing, with a daily processing capacity of 30 tonnes, while samples of the FM group were collected from a facility in Yicheng, Shanxi, operating at a batch size of 90 tonnes. Both processes used cattle manure as the initial materials. Cattle manure was obtained from several local beef cattle farms. The initial carbon-to-nitrogen ratios (C/N) were 15.12 for FC and 16.07 for FM, and the moisture contents were 55.30% and 64.33%, respectively (Tables S1 and S2). In FC, materials were introduced at one end of the trough and mechanically agitated using a self-propelled turner as they progressed toward the discharge end. This stage lasted for 31 days, with turning performed every two days during the initial 14 days and every four days during the following 17 days. Subsequently, the materials were transferred to a curing workshop for approximately 60 days of static secondary maturation (curing). The entire composting process lasted for 91 days. Samples were collected on days 0, 19, 31, and 91 by the quartering method. Briefly, day 0 represented the original material, day 19 represented the transition from the high-temperature phase to the cooling phase, day 31 represented the completion of the first stage, and day 91 represented the end of the curing process. In FM, a molecular semi-permeable membrane was used to cover the compost pile. Aeration was supplied by air blowers, and the flow rate was maintained at 46 m^3^/min. The entire composting process lasted for 31 days. Compost samples were collected on days 0, 10, 19, and 31 by the quartering method. Day 0 represented the original material, day 10 represented the high temperature phase, day 19 represented the transition from the high temperature phase to the cooling phase, and day 31 represented the completion of composting.

The core temperature of the compost pile was measured daily using an industrial thermometer (Figure S1). Both final compost products were used as commercial organic fertilizers. After collection, each sample was divided into two subsamples. One subsample was lyophilized in a freeze dryer for 48 h, ground, and sieved through a 60-mesh screen. The obtained powder was stored at −20 °C for the determination of antibiotics, ARGs, and high-throughput sequencing. The other subsample was kept fresh and stored at 4 °C for physicochemical parameter analysis.

### 2.2. Physicochemical Indicators

5.0 g aliquot of the fresh sample was extracted with 2 M potassium chloride (KCl) solution at a solid-to-liquid ratio of 1:5 (*w*/*v*). The mixture was sealed with parafilm and shaken on an orbital shaker at 200 rpm for 1 h. Subsequently, the extract was centrifuged at 4000 rpm for 20 min, and the supernatant was vacuum-filtered through a 0.45 μm membrane filter to obtain the extract solution. The extract solution was used to measure the concentrations of nitrate-nitrogen (NO_3_^−^-N), using a spectrophotometer [23]. The moisture content (MC) was determined by drying the samples at 105 °C for 24 h [24]. The pH was measured using a pH meter based on a 1:10 (*w*/*w*) suspension of fresh samples in deionized water [25]. The C/N was measured using an elemental analyzer (Vario MICRO cube, Elementar, Langen, Germany). The results for these indicators are shown in Tables S1 and S2.

### 2.3. Antibiotic Analysis

This study investigated sulfonamide and tetracycline antibiotics frequently detected in cattle manure, including sulfamonomethoxine (SMM), sulfadiazine (SD), sulfamerazine, sulfamethazine (SM2), sulfamethoxazole, tetracycline (TC), and chlortetracycline (CTC). The specific treatment protocol was as follows:

Extraction: 1.0 g of sample was mixed with 2.0 mL of deionized water, vortexed, and allowed to stand for 20 min before centrifuging. Then, 5.0 mL of Na_2_EDTA-McIlvaine buffer was added, and the mixture was shaken for 1 min. Subsequently, 10 mL of acidified acetonitrile (2%) and 1 g of NaCl were added. The mixture was then shaken for 1 min, vortexed at 1500 rpm for 10 min, and centrifuged at 5000 rpm for 20 min.

Purification: An 8 mL aliquot of the supernatant was transferred into a new tube and mixed with 0.5 g of anhydrous Na_2_SO_4_, 100 mg of primary secondary amine (PSA), and 50 mg of C_18_. The mixture was shaken for 1 min, vortexed at 1500 rpm for 10 min, and centrifuged at 5000 rpm for 20 min. Subsequently, 5.0 mL of the resulting supernatant was collected, evaporated to dryness under a gentle nitrogen stream, and finally reconstituted in 1.0 mL of a methanol/water mixture (80:20, *v*/*v*). Antibiotics were analyzed using the Agilent 1260 HPLC system (Agilent Technologies, Santa Clara, CA, USA); specific detection conditions can be found in Table S3.

### 2.4. ARGs and Bacterial Community Analysis

Total DNA was extracted from the samples using the Fast DNA SPIN Kit for Soil (MP Biomedicals, Santa Ana, CA, USA) following the manufacturer’s protocol. The A_260_/A_280_ ratio and concentration of extracted DNA were determined using a Nano Drop 2000 spectrophotometer (Thermo Scientific, Waltham, MA, USA) and agarose gel electrophoresis (1.0%). Only DNA extracts with an A_260_/A_280_ ratio greater than 1.8 were used for subsequent analyses. Droplet digital PCR (ddPCR) was used to quantify seven tetracycline ARGs (*tetW*, *tetX*, *tetA*, *tetB*, *tetC*, *tetG*, and *tetQ*), three sulfonamide ARGs (*sul1*, *sul2*, and *sul3*) and one trimethoprim resistance gene (*dfrA7*). The inclusion of *dfrA7* was due to its frequent co-occurrence with sulfonamide resistance genes (e.g., *sul1* and *sul2*) in bacteria. In addition, *intI1* was also analyzed as an indicator of the horizontal transfer potential of ARGs. For each ddPCR run, no-template controls and extraction blanks were incorporated. Reaction wells with more than 1 × 10^4^ accepted droplets were considered valid for further analysis. The primers, annealing temperature, and ddPCR reaction conditions were provided in Tables S4 and S5.

### 2.5. Bacterial Community Analysis

Genomic DNA was first extracted from each sample, and DNA integrity was subsequently verified by agarose gel electrophoresis. Next, barcoded primers targeting the region of interest (e.g., the 16S rRNA gene) were designed and synthesized, followed by PCR amplification using TransStart FastPfu DNA Polymerase (TransGen Biotech, Beijing, China). The resulting amplicons were then purified by agarose gel electrophoresis and gel extraction, quantified using the QuantiFluor™-ST blue fluorescence quantification system (Promega, Madison, WI, USA), and normalized for library preparation. Thereafter, a PE300 sequencing library was constructed through Y-shaped adapter ligation, magnetic bead purification, and PCR enrichment, after which high-throughput sequencing was performed on the Illumina PE300 platform (Shanghai, China). Specifically, 16S rRNA gene sequencing was performed by Linen Biotechnology Co., Ltd. (Shanghai, China). The V3–V4 region of the 16S rRNA gene was amplified using the 341F and 806R primer pairs. The PCR products were analyzed via 1.0% agarose gel electrophoresis, and the target fragments were subsequently recovered and quantified using the QuantiFluor™-ST system (Promega, Madison, WI, USA). The purified amplicons were sequenced on an Illumina MiSeq platform using the PE300 strategy.

After sequencing, raw reads were quality-filtered, and chimeric sequences were subsequently removed. The clean reads were then clustered into operational taxonomic units (OTUs) at 97% sequence similarity or, alternatively, resolved into amplicon sequence variants (ASVs) using the DADA2 pipeline. Consequently, an OTU/ASV abundance table was generated. Based on this table, a series of bioinformatic analyses were carried out, including alpha diversity assessment (e.g., Shannon and Simpson indices), taxonomic composition profiling, functional prediction based on 16S rRNA gene sequences, and other relevant analyses.

### 2.6. Data Analysis

Both absolute and relative abundances were used to analyze the dynamic changes in ARGs. Absolute abundance of ARGs and *intI1* was calculated as:

(1) $${C_{i}^{j}}_{\left(abs\right)}=\frac{C_{ddPCR}\times V_{PCR}\times V_{DNA}\times DF}{W_{sample}}$$

where

${C_{i}^{j}}_{\left(abs\right)}$: the absolute abundance of the *j*-th target (ARGs and *intI1*) in the *i*-th sample, expressed as copies/g fresh weight;

$C_{ddPCR}$: the concentration measured by digital PCR (copies/μL);

$V_{PCR}$: the volume of the dPCR reaction system (μL);

$V_{DNA}$: the final volume of extracted DNA (μL);

*DF*: The dilution factor of sample DNA prior to ddPCR;

$W_{\mathrm{sample}}$: the fresh weight of the sample used for DNA extraction (g).

The relative abundances of ARGs and *intI1* were calculated by normalization to the 16S rRNA gene absolute abundance. The formula is as follows:

(2) $${C_{i}^{j}}_{\left(rel\right)}=\frac{{C_{i}^{j}}_{\left(abs\right)}}{{C_{i}^{16s}}_{\left(abs\right)}}$$

where

${C_{i}^{j}}_{\left(rel\right)}$: the relative abundance of the *j*-th target ARG in the *i*-th sample (copies per copy of 16S rRNA gene);

${C_{i}^{16s}}_{\left(abs\right)}$: the absolute abundance of the 16S rRNA gene in the *i*-th sample (copies/g fresh weight).

All experiments were performed in triplicate. Data were processed using Microsoft Excel 2019, and one-way analysis of variance (ANOVA) was conducted using IBM SPSS Statistics 26.0 for significance analysis, with *p* < 0.05 considered statistically significant. Column charts were generated using Origin 2021. Co-occurrence network analysis was performed to explore correlations between bacteria and ARGs. Spearman’s correlation was calculated between bacterial and ARGs datasets, and Benjamini–Hochberg (FDR) correction was applied for multiple testing. A stringent threshold of r > 0.8 and *p* < 0.05 was used to filter strong correlations. All above procedures were performed in R software (v4.5.1) using the “psych” (v2.4.6) package, followed by network visualization and analysis in Gephi (0.9.2). All data were Z-standardized prior to further multivariate analysis. Heatmaps, redundancy analysis (RDA), Mantel test, and PLS-PM were performed in R software using the “pheatmap” (v1.0.12), “vegan” (v2.6-4), “ggplot2” (v3.5.1), and “plspm” (v0.5.1) packages, respectively.

## 3. Results and Discussion

### 3.1. Changes in Antibiotics During Composting

**Figure 1:**
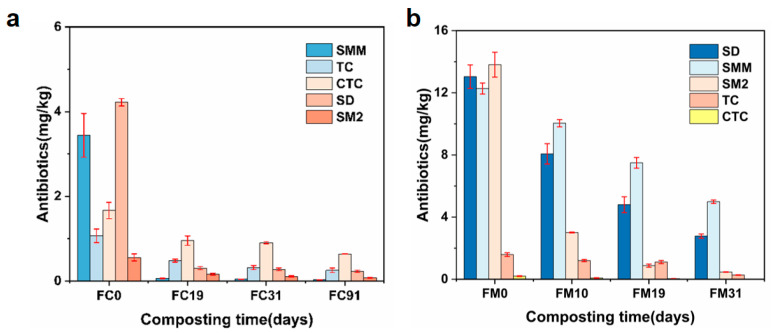
Concentrations of five antibiotics detected during push-flow trough composting (FC) (**a**) and membrane-covered composting (FM) (**b**). SMM: sulfamonomethoxine; SD: sulfadiazine; SM2: sulfamethazine; TC: tetracycline; CTC: chlortetracycline.

As shown in Figure 1, five target antibiotics were detected in both sample groups, including SMM, SD, SM2, TC and CTC. Due to variations in animal husbandry practices and medication usage across regions, the distribution of antibiotics in cattle manure differed. In the initial sample, the total antibiotic concentration in FC was 11.15 mg/kg, dominated by SD (4.28 mg/kg) and SMM (3.44 mg/kg). The initial sample in FM exhibited a higher antibiotic concentration of 40.91 mg/kg, dominated by SD (13.04 mg/kg), SM2 (12.27 mg/kg), and CTC (13.81 mg/kg). These high detection levels suggest that cattle manure is a significant reservoir of antibiotics.

After composting, the total antibiotic removal rates were 88.89% (FC) and 79.20% (FM) (Figure 1). Specifically, the FC group exhibited high removal rates for sulfonamide antibiotics. For SM2, SD, and SMM, removal rates reached 85.62%, 94.16%, and 99.13%, respectively. This performance can be attributed to the rapid heating profile in FC, which reached 65 °C on day 5, with the cumulative thermal dose peaking at 69 °C·day on day 10 (Figures S1 and S3). Such conditions promoted the abiotic pyrolysis and hydrolysis of antibiotics [26]. Concurrently, the enhanced aeration derived from mechanical turning bolstered microbial metabolic activity, facilitating the biotic degradation of these pollutants [27]. However, the FC group demonstrated relatively low removal rates for tetracycline antibiotics, with removal rates of 77.31% for TC and 64.68% for CTC. This may be related to the generally greater biodegradability of sulfonamide antibiotics compared to tetracyclines [28]. In comparison, the FM group demonstrated superior comprehensive antibiotic removal capabilities. It achieved removal rates of 82.77%, 78.68%, 96.64%, 100%, and 59.34% for TC, SD, CTC, SMM, and SM2, respectively. These outcomes were associated with the prolonged high-temperature phase, high moisture content, and effective nitrogen retention. Furthermore, compost piles consistently maintained an alkaline environment (FC: pH = 7.68–8.60; FM: pH = 7.65–8.82), which also favored antibiotic removal [29]. Nevertheless, the removal rates of SM2 and SD in FM were slightly lower than those in FC. This may be because the dynamic turning and continuous advancement in FC facilitated the contact between oxygen and materials, while large-scale static composting in FM may have formed partial anaerobic environments. In addition, Figure 1 also reveals that the removal of antibiotics in both processes predominantly occurred in the high-temperature stage (0–19 d). This phase removed 81.47% (FC) and 65.01% (FM) of total antibiotics. In the cooling phase (19–31 or 91 d), the total removal rates only increased by 7.43% (FC) and 14.19% (FM), highlighting the critical role of temperature in antibiotic removal. Overall, both treatments demonstrated significant antibiotic removal capabilities.

### 3.2. Changes in ARGs and intI1 During Composting

**Figure 2:**
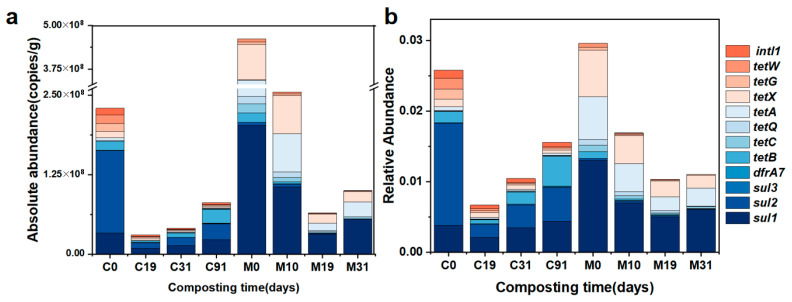
Changes in the absolute (**a**) and relative abundance (**b**) of ARGs and class 1 integrons (*intI1*) during push-flow trough composting (FC) and membrane-covered composting (FM).

Figure 2a shows the changes in the absolute abundance (AA) of ARGs and *intI1* during composting. In the raw compost materials, the total abundance of ARGs was 2.19 × 10^8^ copies/g (FC) and 3.54 × 10^8^ copies/g (FM), with *sul2* and *sul1* being the predominant ARGs. Specifically, the AA of *sul1* was 3.37 × 10^7^ copies/g in FC and 1.97 × 10^8^ copies/g in FM. The AA of *sul2* was 1.29 × 10^8^ copies/g in FC and 9.50 × 10^7^ copies/g in FM. These high abundances were consistent with the widespread use of sulfonamide antibiotics in animal feeds [30].

Besides *sul1* and *sul2*, the four dominant genes in FC were *tetB* (1.42 × 10^7^ copies/g), *tetW* (1.38 × 10^7^ copies/g), *tetG* (1.25 × 10^7^ copies/g), and *tetX* (9.51 × 10^6^ copies/g). The four dominant genes in FM were *tetW* (1.50 × 10^7^ copies/g), *tetQ* (1.44 × 10^7^ copies/g), *tetB* (1.25 × 10^7^ copies/g), and *tetX* (8.49 × 10^6^ copies/g). These results indicate a consistency in ARG abundance across samples from different regions, which could provide a basis for the reduction in ARGs in cattle manure. Simultaneously, the initial AA of *intI1* reached 1.01 × 10^7^ copies/g in FC and 1.02 × 10^8^ copies/g in FM, suggesting the potential for horizontal gene transfer (HGT).

Relative abundance (RA) can mitigate errors induced by sampling and operational variations [31]. As shown in Figure 2b, during the high-temperature phase (days 0–19), the total RAs of ARGs and *intI1* decreased significantly and dropped to their minimum values. The total removal rates of ARGs and *intI1* reached 74.01% in FC and 63.16% in FM. This suggests that high temperature is effective at killing bacterial hosts or destroying plasmids [32]. In Figure S2, MC and C/N exhibited strong positive correlations with *intI1* (*p* < 0.01), suggesting that they may directly drive *intI1* degradation. This may be because C/N is a critical factor shaping microbial community structure. And the suitable moisture conditions could facilitate microbial metabolism and improve the efficiency of HGT [33]. Notably, a clear rebound of ARGs was observed in FC during the cooling phase (19–91 d). The RA of total sulfonamide resistance genes increased from 4.00 × 10^−3^ to 9.29 × 10^−3^ and that of total tetracycline resistance genes increased from 2.14 × 10^−3^ to 5.56 × 10^−3^. This phenomenon may result from three potential causes: (1) Since ARGs predominantly reside in mesophilic or psychrophilic microorganisms, the high temperature severely suppressed their survival and metabolism, while the cooling phase facilitated their recovery [34]. (2) The atmospheric environment of composting plants harbors diverse ARGs and MGEs. The rebound of ARGs in the late composting stage might be related to aerosol contamination [35]. (3) Following a reduction to a minimum on day 19 (5.36 × 10^−4^), *intI1* also rebounded during the cooling phase (2.03 × 10^−3^ on day 91), suggesting that ARG dynamics are correlated with the HGT process [36]. In addition, *sul1* and *sul2* exhibited the largest rebound quantities, at 2.29 × 10^−3^ and 2.89 × 10^−3^ (19–91 d), respectively. That may be related to *sul1* being a component of *intI1* [37] and *sul2* frequently residing on plasmids [38].

In contrast, the FM process effectively mitigated this rebound. Only a few ARGs (*sul2*, *sul1*, *tetQ*, *tetA*) showed a rebound from day 19 to day 31, and *tetW*, *tetG*, *tetX*, and *tetC* achieved 100% removal rates on day 31 (Figure 2b). This could be attributed to the superior thermal retention properties of the FM group, which maintained pile temperatures above 50 °C for 15 days, with the cumulative thermal dose reaching its maximum of 54 °C·day on day 19 (Figures S1 and S3). The extended thermophilic period facilitated the elimination of bacterial hosts. Another potential reason is that the semipermeable membrane blocked bioaerosols containing abundant ARGs and MGEs from the ambient air [39], thus preventing a rebound in ARG abundances during the cooling phase.

On day 31, the FC group achieved removal rates of 59.97% for ARGs and 2.31% for *intI1*, while the FM group achieved 76.11% and 69.13% (Figure 2b). However, by the end of the curing phase (day 91) in FC, the removal rates for ARGs and *intI1* decreased to 39.49% and −78.87%, respectively. Although the removal effects of ARGs and *intI1* in FM appear stronger than those in FC, it should be noted that the FM compost did not undergo an extended aging period; whether ARGs will increase significantly in the subsequent storage process remains to be studied. From a practical standpoint, these results indicate that long-term storage of compost products should be avoided due to the risk of ARGs increasing [40].

### 3.3. Bacterial Community Succession During Composting

**Figure 3:**
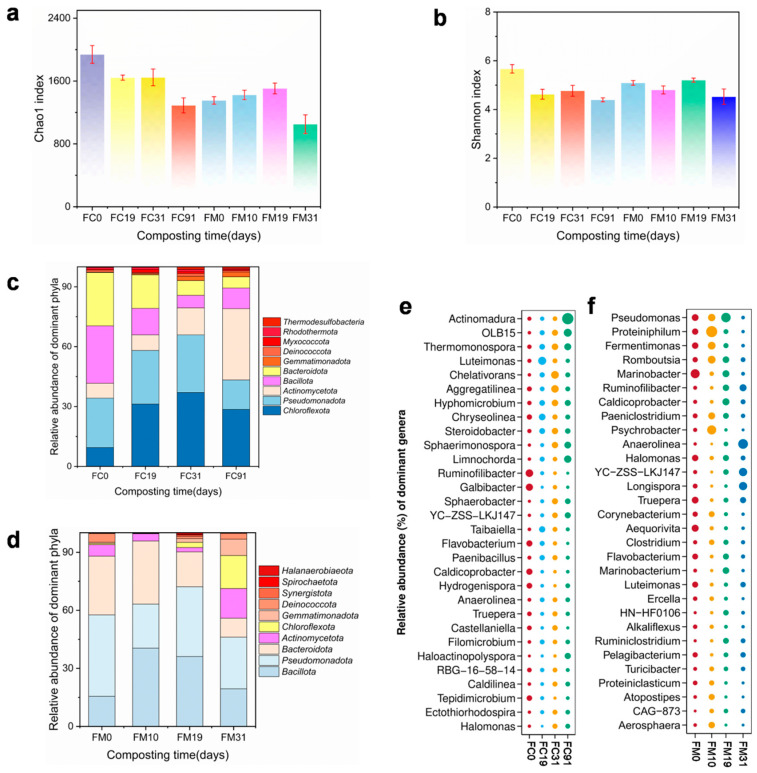
Changes in bacterial community during cattle manure composting. Changes in the Chaol index (**a**) and the Shannon index (**b**). Relative abundance of the top 10 phyla during push-flow trough composting (**c**) and membrane-covered composting (**d**). Relative abundance of the top 30 genera after Z-standardization during push-flow trough composting (**e**) and membrane-covered composting (**f**) with larger dots indicate a higher relative abundance of the species.

Figure 3a,b illustrate the changes in bacterial community α-diversity during composting. Due to the nutrient shifts in the compost piles, both treatments exhibited a declining trend in α-diversity [41]. Specifically, the Chao1 index in FC decreased from 1938.72 (day 0) to 1290.87 (day 91), and the Shannon index decreased from 5.67 (day 0) to 4.40 (day 91). Despite fluctuations on day 31, the overall decline remained significant. Similarly, in FM, the Chao1 index decreased from 1353.58 (day 0) to 1051.70 (day 31), and the Shannon index dropped from 5.09 (day 0) to 4.53 (day 31). Notably, these indices rebounded during the middle composting phase, potentially due to temperature fluctuations stimulating microbial responses [42].

In Figure 3c, five phyla dominated the entire composting process. These phyla are *Bacillota*, *Pseudomonadota*, *Bacteroidota*, *Chloroflexota*, and *Actinomycetota*, which consistently exceeded 90% in FC and FM. Among these phyla, the proportion of *Bacillota* in FC decreased from 27.44% (day 0) to 6.11% (day 31), followed by a slight increase to 10.12% (day 91). In contrast, the proportion of *Bacillota* was strongly enriched in FM, increasing from 15.11% (day 0) to 39.89% (day 31) and then to 35.43% (day 91). This difference was associated with the characteristics of the two processes. The FC group had a short duration of high temperatures, while the FM group maintained a prolonged high temperature period. Because *Bacillota* is rich in thermophilic genera capable of producing spores to survive temperature stress [43], these genera successfully occupied a dominant ecological niche in FM. *Pseudomonadota* constituted another major dominant phylum, accounting for 23.66% (FC) and 40.82% (FM) of the initial samples. Due to its high metabolic flexibility [44], it consistently maintained high proportions in both processes, ranging from 14.46% to 28.21% in FC and from 22.51% to 40.82% in FM. *Bacteroidota*, a typical enteric microorganism, dominated the early composting phase, with proportions ranging from 16.41% to 25.55% (FC) and from 17.71% to 32.03% (FM) during the first 19 days. This dominance is attributed to its ability to enhance digestion and utilize carbohydrates [45]. However, as the composting progressed, dissolved organic matter was gradually consumed. The survival advantage of *Bacteroidota* declined, ultimately decreasing to 5.47% (FC, day 91) and 9.53% (FM, day 31).

In contrast, *Actinomycetota* demonstrated a prominent competitive advantage during the later phase of composting. In FC, its proportion increased from 7.14% (day 0) to 35.08% (day 91). In FM, its initial proportion was 5.61%. Although it experienced slight declines on day 10 (3.71%) and day 19 (2.20%), it still increased to 14.74% on day 31 (Figure 3c). This is because *Actinomycetota* can decompose complex organic matter (such as cellulose and lignin), promoting the transformation of materials into mature fertilizer in the later stage of composting [46]. Similarly, since *Chloroflexota* can generate energy through photosynthesis [47], it gains a survival advantage in nutrient-poor environments. Although its initial proportions were low in both groups (FC: 9.03%, FM: 0.70%), it ultimately increased to 28.00% (FC) and 16.51% (FM) (Figure 3c). In general, apart from *Bacillota*, no notable differences were observed in the bacterial evolutionary trends between the two groups.

Figure 3e–f illustrate the evolution of bacterial communities at the genus level. In the initial samples, the dominant genera in FC were *Ruminofilibacter* (22.02%), *Galbibacter* (18.40%), *Caldicoprobacter* (11.31%), *Flavobacterium* (10.47%), *Hydrogenispora* (8.26%), *Chryseolinea* (6.14%), and *Aggregatilinea* (4.61%). In FM, the dominant genera included *Marinobacter* (21.48%), *Pseudomonas* (8.87%), *Truepera* (8.79%), *Fermentimonas* (7.42%), *Halomonas* (7.24%), and *Proteiniphilum* (6.85%). After composting, the proportions of *Actinomadura*, *OLB15*, and *Thermomonospora* in FC increased by 15.93-fold, 14.66-fold, and 1.86-fold, respectively. The proportions of *Anaerolinea*, *YC-ZSS-LKJ147*, and *Ruminiclostridium* in FM were enriched by 130.32-fold, 15.39-fold, and 17.72-fold, respectively. The enrichment of these genera implies their potential contribution to the compost maturation. Among these genera, *Anaerolinea* is a strictly anaerobic bacterium capable of degrading organic matter in oxygen-depleted zones [48]. Its abundance increased by 1.30-fold in FC but by 130.32-fold in FM, suggesting that the larger scale of the FM compost resulted in uneven oxygen distribution, thus fostering partial anaerobic environments. All the above findings demonstrate that the variations in ARGs within the composting system were closely linked to the microbial communities.

### 3.4. Relationship Between ARGs, MGE and Bacterial Community

**Figure 4:**
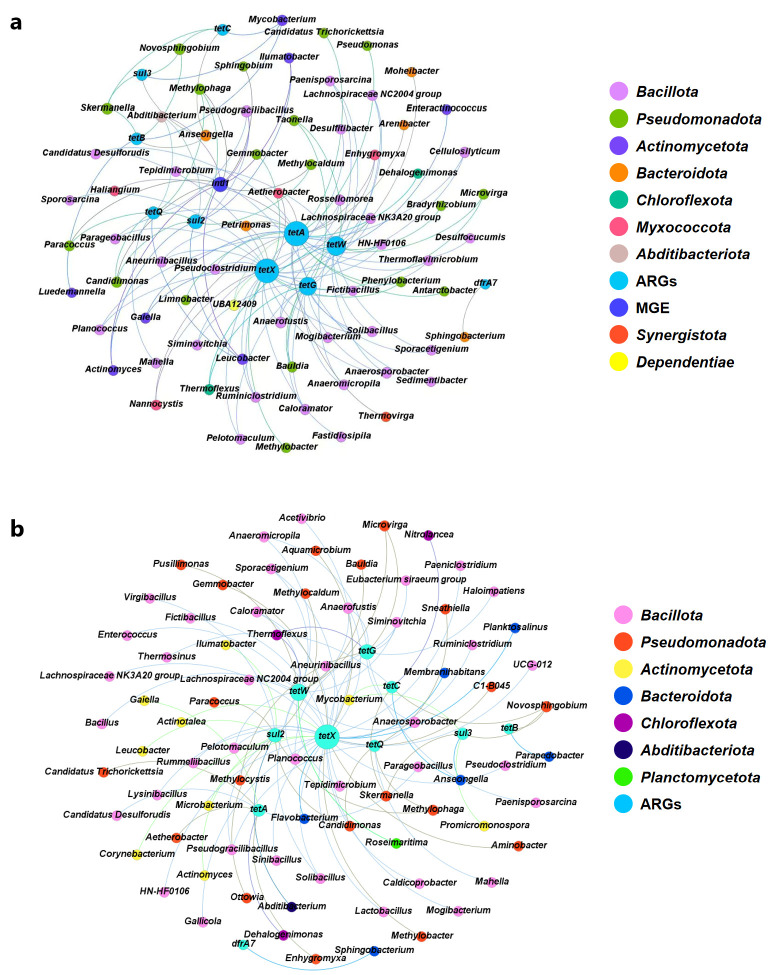
The co-occurrence network revealing relationships between ARGs, *intI1* and bacterial genera in FC (**a**) and FM (**b**) (r > 0.8, *p* < 0.05). Node color represents the type (ARGs or phylum), and node size is determined by the degree in network.

To investigate the co-occurrence patterns between ARGs, *intI1* and bacterial genera, a network analysis was performed (r > 0.8, *p* < 0.05). Table S6 shows that compared with the FM group, the FC group had a higher average degree and network density and lower average path length, which indicates closer interactions between microorganisms and ARGs in FC [49]. A total of 70 (FC, Figure 4a) and 82 (FM, Figure 4b) bacterial genera were significantly associated with ARGs and *intI1*, and 46 bacteria genera were shared in both groups. Bacteria genera showing the strongest co-occurrence with ARGs and *intI1* were mainly distributed in *Bacillota* and *Pseudomonadota*. *Bacillota* accounted for as high as 44.28% (FC) and 48.71% (FM) of the total strongly co-occurring taxa, and *Pseudomonadota* accounted for 25.71% (FC) and 23.08% (FM) of those. There were also 7.15% (FC) and 7.70% (FM) taxa distributed in *Bacteroidota*, and 2.86% (FC) and 3.84% (FM) taxa distributed in *Chloroflexota*. Meanwhile, it was found that ARGs co-occurred with the same bacteria. For example, *Anaerofustis* showed strong co-occurrence with *tetX*, *tetG*, and *tetW*, and *Candidimonas* exhibited significant co-occurrence with *sul2* and *tetQ*. This phenomenon explains the simultaneous presence of multiple ARGs in the environment and accounts for the ability of a single antibiotic to concurrently enhance the abundance of various ARGs. Furthermore, there were nine genera related to *intI1* in FC, including *Abditibacterium*, *Candidimonas*, *Leucobacter*, *Methylophaga*, *Pseudogracilibacillus*, *Actinomyces*, *Ilumatobacter*, *Methylocaldum*, and *Sphingobium*. These genera (except *Sphingobium*) were strongly associated with both sulfonamide and tetracycline resistance genes, indicating them as potential risk vectors for the horizontal transfer of ARGs. However, in FM, no genera showed significant correlation with *intI1*, suggesting that it may have spread widely in the community, thereby preventing its precise localization by statistical methods. This phenomenon also implies that the *intI1*-mediated HGT process may be widely occurring in the FM group.

According to the global health risk assessment for ARGs, *tetQ* has the highest risk of human exposure [50]. Figure 4 shows that *tetQ* was strongly associated with *Abditibacterium*, *Candidimonas*, *Leucobacter*, *Methylophaga*, and *Pseudogracilibacillus* in FC and with *Candidimonas*, *Mahella*, *Pseudogracilibacillus*, *Methylophaga*, and *Sneathiella* in FM. These genera, which include both aerobic and facultative anaerobic bacteria, are well-suited to stressful environments. Specifically, *Leucobacter* exhibits high chromate tolerance and a wide pH growth range (pH = 5–10) [51]. *Mahella* can maintain activity at high temperatures (up to 60 °C) and tolerate NaCl concentrations as high as 4%. *Candidimonas* is a denitrifying bacterium, exhibiting strong adaptability to nitrate-rich agricultural soils [52]. *Methylophaga* exhibits halophilic and alkalitolerant characteristics [53]. In addition, *Candidimonas* and *Methylophaga* showed strong co-occurrence with *tetQ* in both groups, indicating that controlling specific bacteria genera may be a strategy to reduce the risk of ARGs.

### 3.5. Relationship Between ARGs, Antibiotics, Environmental Factors and Bacterial Community

**Figure 5:**
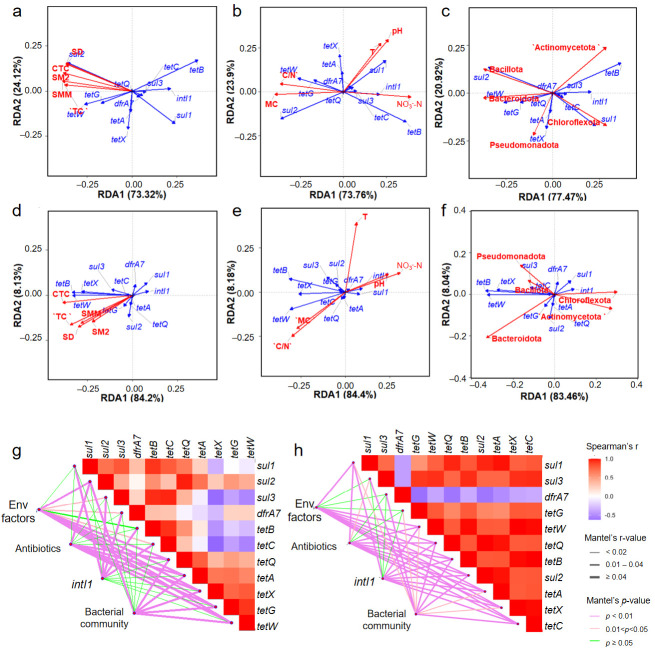
Redundancy analyses between antibiotics (**a**), environmental factors (**b**), bacterial community (**c**) and ARGs (blue lines) in FC. Redundancy analysis between antibiotics (**d**), environmental factors (**e**), bacterial community (**f**), and ARGs in FM. Environmental factors include temperature (T), moisture content (MC), pH, nitrate nitrogen (NO_3_^−^-N), and carbon to nitrogen ratio (C/N). Bacterial community includes major phylum-level microorganisms. Mantel test analyses of ARGs with environmental factors (env factors), antibiotics, *intI1*, and bacterial community in FC (**g**) and FM (**h**). The thickness of the lines represents the Mantel correlation (r value) between indicators and ARGs, while the color gradient ranging from green to purple indicates the significance level (*p* value).

RDA was used to investigate the relationships between environmental factors (pH, T, MC, C/N, NO_3_^−^-N), antibiotics, bacterial community (based on the phyla level) and ARGs. As shown in Figure 5a–f, the sum of RDA1 and RDA2 exceeded 90% and was statistically significant. Figure 5a and Figure 5d depict the effects of antibiotics on ARGs in the FC and FM groups, respectively. In FC, antibiotics were positively correlated with *tetG*, *tetW*, and *sul2*. In FM, antibiotics were positively correlated with more ARGs, including all tetracycline resistance genes, *sul2*, and *sul3*. These results indicated that antibiotics exerted selective pressure on ARGs during composting. Figure 5b,e showed the RDA of environmental factors and ARGs. Temperature exhibited a greater explanatory capacity for ARG variation than other environmental factors, owing to its direct inhibition of host bacteria and the degradation of antibiotics. Additionally, environmental factors such as nitrogen availability, pH levels, and moisture content influenced ARGs by impacting the survival of host bacteria. Optimal bacterial growth and metabolism require adequate nitrogen levels, while extreme pH values are detrimental to bacterial survival. Furthermore, most microorganisms thrive in environments with approximately 55–60% moisture content. Since the relatively dry environment in FC was not conducive to the growth of some hosts, moisture (MC) was negatively correlated with most ARGs (Tables S1 and S2). In addition, bacterial phyla also exhibited significant influence on the variation in ARGs. In FC, *Chloroflexota* and *Actinomycetota* were positively correlated with *tetB*, *sul3*, *dfrA7*, *intI1*, *tetC*, and *sul1*. *Bacillota* was positively correlated with *sul2*, *tetW*, *tetG*, and *tetQ* (Figure 5c). In FM, *Chloroflexota* and *Actinomycetota* were positively linked to *intI1*, *sul1* and *dfrA7*. *Bacillota* was positively linked to *sul2*, *sul3*, *dfrA7*, *tetB*, *tetW*, *tetX*, and *tetC* (Figure 5f). These results indicated that microorganisms played a potential role in the carriage and dissemination of ARGs.

Mantel tests further confirmed the significant influence of environmental factors, antibiotics, and bacterial community on ARG variation (Figure 5g,h). Meanwhile, it was found that *intI1* was correlated with ARGs. In FC, *intI1* was strongly correlated with *sul1*, *sul2*, *sul3*, *tetB*, and *tetC* (r = 0.65–0.95, *p* < 0.05). In FM, *intI1* was strongly correlated with all ARGs except *dfrA7* (r = 0.79–0.99, *p* < 0.01). These results suggested that the above ARGs might be transmitted through the *intI1*-mediated HGT process. Although the correlation between *dfrA7* and *intI1* was weak, it may also be captured by other mobile elements (such as second-class integrons or transposons), and then experience the HGT process between microorganisms. High temperatures in the thermophilic phase lyse bacterial cells and release extracellular ARGs (eARGs). The degradation rate of extracellular DNA (eDNA) has a nonlinear convex relationship with pH, peaking at around pH 8. eDNA released via high-temperature bacterial lysis has a short half-life and is readily degraded by extracellular nucleases [54]. In this study, pH values were 7.68–8.82 in FC and 7.96–8.88 in FM, which facilitated relic DNA degradation. Furthermore, high temperature disrupts the DNA double-helix structure, and liberated genetic fragments are efficiently broken down by extracellular nucleases [55]. In addition, high-temperature composting could shorten the half-life of ARGs from 3.9 days in conventional composting to 1.3 days [56]. These findings indicate that the detected *intI1* correlations may stem from active HGT in viable biofilms, rather than passive relic DNA retention.

Furthermore, Mantel tests showed co-occurrence patterns among ARGs in both treatments. The correlation strength of ARGs in FM was higher than that in FC, which may be due to the more stable composting environment in FM. In contrast, the frequent turning in FC created fluctuating conditions (e.g., temperature and humidity fluctuations, air pollution), which inhibited mobile element activity, thereby reducing the probability of gene co-transmission.

**Figure 6:**
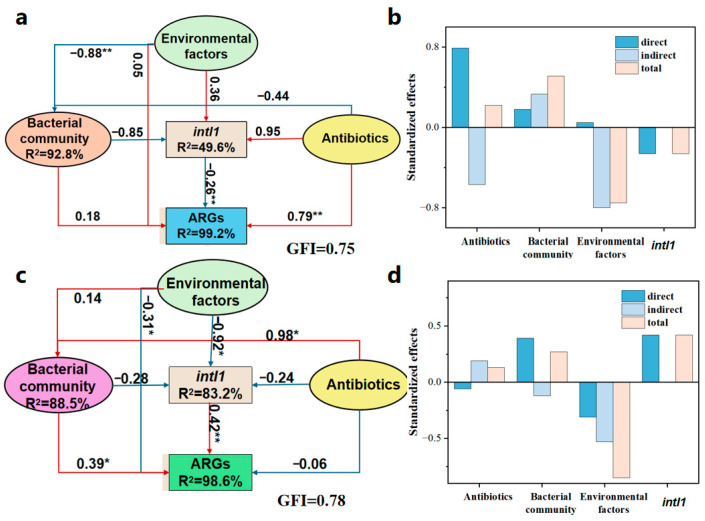
Partial Least Squares Path Model (PLS-PM) reflects the relationships among antibiotics, environmental factors, bacterial community, *intI1*, and ARGs in FC (**a**) and FM (**c**). (**b**,**d**) display the diagrams of standardized total, direct, and indirect effects for FC and FM, respectively. Antibiotic indicators included TC, CTC, SD, SM2, and SMM. Environmental factors included T, pH, NO_3_^−^-N, MC, and C/N. Bacterial community indicators included the Chao1 index, Shannon index, and the relative abundance of the top five phyla. Red and blue arrows represent positive and negative correlations, respectively. Numeric values adjacent to the arrows denote the standardized path coefficients, indicating the magnitude of the relationships. Significance levels are represented by * (*p* < 0.05) and ** (*p* < 0.01). GFI stands for the model’s goodness of fit. Standardized total, direct, and indirect effects of various factors on ARGs are shown in (**b**) (FC) and (**c**) (FM).

PLS-PM analysis provides a more precise quantification of the relationships of individual indicators. As shown in Figure 6a and Figure 6c, these models demonstrated high explanatory power, accounting for 99.2% (FC) and 98.6% (FM) of the variance in ARGs, respectively. Based on the standardized total effect analysis, the bacterial community had the greatest impact on ARG variation in FC (λ = 0.51) (Figure 6b). This was because the bacterial community could directly regulate the vertical gene transfer (VGT) process of ARGs. This result also indicated that the VGT process may be the main transmission mechanism of ARGs in FC. In comparison, the bacterial community and *intI1* were identified as the most influential factors of ARG variation in FM (λ = 0.27 and 0.42, Figure 6d), demonstrating that the two composting treatments operated through different mechanisms to influence ARGs. Although *intI1* exhibited a significant positive effect on ARGs in FM (Figure 6b), it showed a negative effect on ARGs in FC (Figure 6d). This may be due to the stable surface environment promoting intercellular contact. Meanwhile, on the surface of stable solid particles, microorganisms are more likely to form thick and structurally intact biofilms, which create favorable conditions for the HGT process of ARGs [57]. In addition, Figure 2 shows that the AA of *intI1* in FM (1.02 × 10^8^) is much greater than that in FC (1.01 × 10^7^). The network diagram (Figure 4b) shows that *intI1* was not associated with specific genera in FM. All findings reflect the high activity and substantial diffusion risk of *intI1* within the FM process. Based on these findings, novel technologies can be developed to further mitigate ARG dissemination. For instance, functionalized membranes embedded with antimicrobial or quorum-quenching agents may disrupt biofilm formation [58], and the co-application of engineered adsorbents (e.g., modified biochar, clay minerals) during composting could sequester extracellular DNA and immobilize ARGs [59,60]. For antibiotics, they exerted weak positive effects on ARG dynamics, with total effects of 0.22 in FC and 0.13 in FM. Notably, antibiotics affected ARGs via a direct pathway in FC, whereas in FM, this influence was indirect, acting mainly through the bacterial community. These models also found that environmental factors negatively affected ARGs in both systems. In FC, this effect was indirectly mediated through the bacterial community, while in FM, it was channeled primarily through *intI1* (Figure 6). In general, all results suggested that the dissemination and variation in ARGs were affected by the type of composting.

## 4. Conclusions

This study demonstrates that the FM group more effectively prevented the rebound of ARGs and *intI1* during the cooling phase and enhanced their removal compared to the FC group. This outcome may be attributed to prolonged thermophilic conditions, shifts in bacterial community composition, and reduced aerosol contamination in FM. PLS-PM analysis indicated that the bacterial community had the greatest impact on ARG variation in FC, while in FM, both the bacterial community and the HGT process mediated by *intI1* may be the main drivers. Although the FM group facilitated the removal of ARGs, it may also enhance the risk of *intI1*-mediated horizontal transfer of residual ARGs. Long-term monitoring of ARGs during storage and after field application is essential to assess their ecological risks and to further elucidate the mechanisms underlying ARG dissemination and evolution.

## Data Availability

The original contributions presented in this study are included in the article/Supplementary Materials. Further inquiries can be directed to the corresponding authors.

## Supplementary Materials

The following supporting information can be downloaded at: https://www.mdpi.com/article/10.3390/toxics14050428/s1, Figure S1. Temperature variations in push-flow trough composting (FC) and membrane-covered composting (FM). Figure S2. Pearson correlation analysis between *intI1* and environmental factors during the process of FM. Figure S3. Changes in the cumulative thermal dose during the process of FC and FM. Table S1. Environmental factors in different stages of push-flow trough composting. Table S2. Environmental factors in different stages of membrane-covered composting. Table S3. Antibiotic detection conditions. Table S4. Primer sequences for antibiotic resistance genes. Table S5. Droplet Digital PCR (ddPCR) reaction conditions. Table S6. Parameters of the co-occurrence network.
