# Supplementary material for: The Fate of Antibiotic Resistance Genes and Their Influential Factors During Large-Scale Cattle Manure Composting
Source: Toxics. 2026 May 13;14(5):428. doi: 10.3390/toxics14050428 (PMC13211389; doi:10.3390/toxics14050428)
Supplement: Supplementary file 1 [file toxics-14-00428-s001.zip › toxics-4265850-supplementary-new.pdf]

Supplementary Materials for

The fate of antibiotic resistance genes and their influential factors during large-scale cattle manure composting

Figures: 3.

Tables: 6.

Contents:

Figure S1. Temperature variations in push-flow trough composting (FC) and membrane-covered composting (FM).

Figure S2. Pearson correlation analysis between *intI1* and environmental factors during the process of FM.

Figure S3. Changes in the cumulative thermal dose during the process of FC and FM

Table S1. Environmental factors in different stages of push-flow trough composting.

Table S2. Environmental factors in different stages of membrane-covered composting.

Table S3. Antibiotic detection conditions.

Table S4. Primer sequences for antibiotic resistance genes.

Table S5. Droplet Digital PCR (ddPCR) reaction conditions.

Table S6. Parameters of the co-occurrence network.

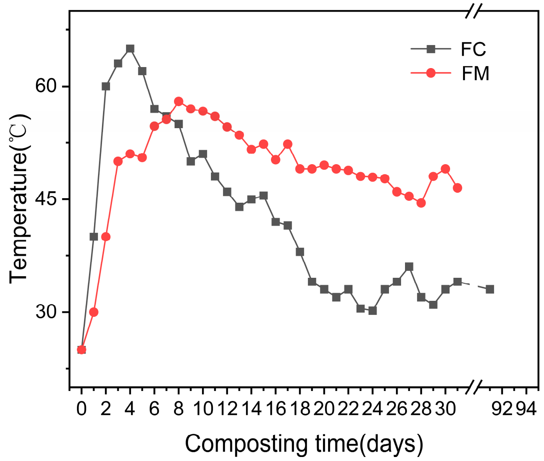

Figure S1. The temperature variations in push-flow trough composting (FC) and membrane-covered composting (FM).

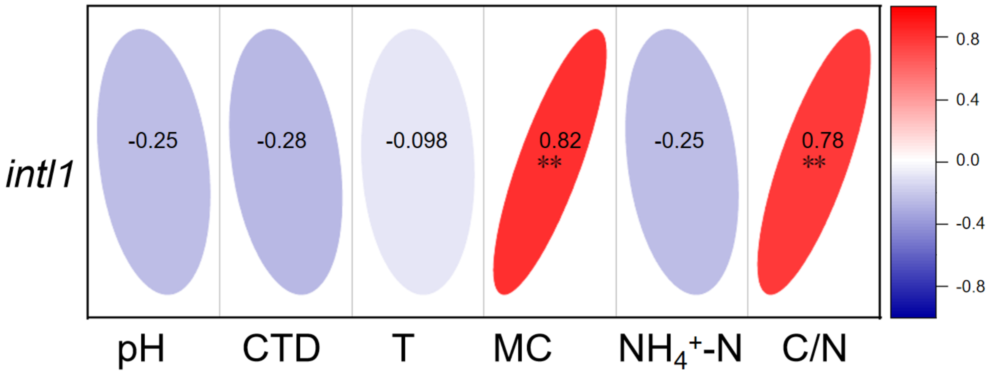

Figure S2. Pearson correlation analysis between *intI1* and environmental factors during the process of FM. The color gradient represents the correlation coefficient, with red indicating positive correlations and blue indicating negative correlations. Asterisks denote significant correlations: *, $p < 0.05$; **, $p < 0.01$; ***, $p < 0.001$; CTD, cumulative thermal dose; T, temperature; MC, moisture content; NH4+-N, ammonium nitrogen; C/N, carbon-to-nitrogen ratio.

$$CTD = \sum (T_i - T_0) \times \Delta t \tag{S1}$$

where:

CTD： the cumulative thermal dose at time i；

Ti： the compost temperature at time i；

T_0： the thermal inactivation threshold for pathogens or ARGs in compost, set at 50°C；

Δt： the duration of Ti。

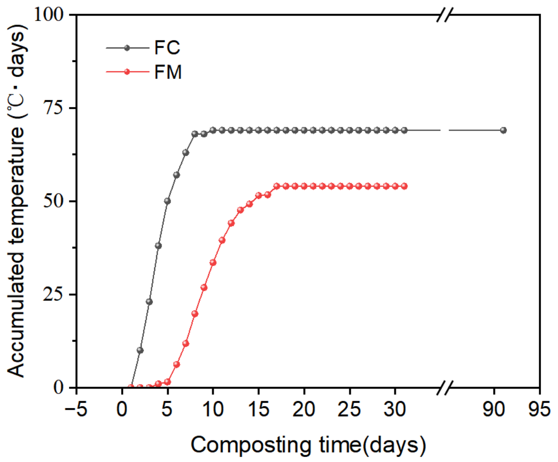

Figure S3. Changes in the cumulative thermal dose during the process of FC and FM

T_0 was set to 50℃, as this is the internationally accepted minimum effective high-temperature threshold for composting. Above this temperature, bacterial host cells undergo significant lysis, directly influencing the fate of ARGs. Meanwhile, we have also supplemented the text regarding temperature in the revised manuscript, as follows.

Table S1. The environmental factors in different stages of push-flow trough composting.

pH	C/N	MC (%)	NO_3^--N (g/kg)

FC0	7.68 ± 0.06	15.12 ± 0.16	55.30 ± 0.98	1.31 ± 0.012
FC19	8.82 ± 0.10	14.18 ±0.48	43.30 ± 1.04	2.16 ± 0.06
FC31	8.60 ± 0.04	13.64 ± 0.44	38.17 ± 1.00	2.47 ± 0.20
FC91	8.29 ± 0.15	13.21 ± 0.27	36.10 ± 1.10	2.92 ± 0.04

Table S2. The environmental factors in different stages of membrane-covered composting.

	pH	C/N	MC(%)	NO_3^--N (g/kg)
FM0	7.96 ± 0.23	16.07 ± 0.84	64.33 ± 3.51	1.32± 0.04
FM10	8.30 ± 0.50	10.65 ±0.40	55.67 ± 0.16	1.48 ± 0.09
FM19	8.49 ± 0.64	9.44 ± 0.18	38.17 ± 1.00	1.96 ± 0.18
FM31	8.88 ± 0.52	7.65 ± 0.41	36.10 ± 1.10	2.08 ± 0.10

MC, Moisture Content; NO_3^--N, Nitrate Nitrogen; FC0, FC19, FC31, and FC91 represent the samples collected on day 0, day 19, day 31, and day

during push-flow trough composting (n=3). FM0, FM10, FM19, and FM31 represent the samples collected on day 0, day 10, day 19, and day 31 during membrane-covered composting (n=3).

Table S3. Antibiotic detection conditions

Antibiotic Group	Mobile Phase Composition	Detection Wavelength	Target Compound	Retention Time	References
**Tetracyclines**	0.12M NaH_2PO_4 solution : Acetonitrile (v : v = 83 : 17)	355 nm	Tetracycline (TC)	5.93 min	Chinese standard (DB37/T 3632-2019)
			Chlortetracycline (CTC)	11.42 min	
**Sulfonamides**	0.30% Acetic acid solution : Acetonitrile (v : v = 75 : 25)	270 nm	Sulfadiazine (SD)	4.02 min	Chinese standard (DB42/T 1993.1-2023)
			Sulfamethoxazole (SMM)	6.94 min	

Sulfadimidine (SM2)	5.58 min

Instrumental analysis: Agilent 1260 HPLC system was used to analyze antibiotics. The size of C18 column was 250 mm × 4.6 mm, 5 μm (Agilent). The flow rate was set at 1.0 mL/min, and the injection volume was 20 uL (n=3).

Table S4. Primer sequences for antibiotic resistance genes

Primer name	sequences（5' to 3'）	Annealing temperature (℃)
tetA-F	GCTACATCCTGCTTGCCTTC	55℃
tetA-R	CATAGATCGCCGTGAAGAGG	55℃
tetB-F	TTGGTTAGGGGCAAGTTTTG	55℃
tetB-R	GTAATGGGCCAATAACACCG	55℃
tetC-F	CTTGAGAGCCTTCAACCCAG	55℃
tetC-R	ATGGTCGTCATCTACCTGCC	55℃

tetW-F	GAGAGCCTGCTATATGCCAGC	60℃
tetW-R	GGGCGTATCCACAATGTTAAC	60℃
tetG-F	GCTCGGTGGTATCTCTGCTC	60℃
tetG-R	AGCAACAGAATCGGGAACAC	60℃
tetQ-F	TTATACTTCCTCCGGCATCG	60℃
tetQ-R	ATCGGTTCGAGAATGTCCAC	60℃
tetX-F	CAATAATTGGTGGTGGACCC	56.5℃
tetX-R	TTCTTACCTTGGACATCCCG	56.5℃
sul1-F	CACCGGAAACATCGCTGCA	58℃
sul1-R	AAGTTCCGCCGCAAGGCT	58℃
sul2-F	TCCGGTGGAGGCCGGTATCTGG	58℃
sul2-R	CGGGAATGCCATCTGCCTTGAG	58℃
sul3-F	CCCATACCCGGATCAAGAATAA	58℃

sul3-R	CAGCGAATTGGTGCAGCTACTA	58℃
dfrA7-F	AAATGGCGTAATCGGTAATG	56.5℃
dfrA7-R	GTGAACAGTAGACAAATGAAT	56.5℃
intI1-F	CTGGATTTCGATCACGGCACG	58℃
intI1-R	ACATGCGTGTAAATCATCGTCG	58℃
16S rRNA-F	CCTACGGGAGGCAGCAG	60℃
16S rRNA-R	ATTACCGCGGCTGCTGG	60℃

Table S5. Digital PCR (dPCR) reaction conditions

Reaction premix system	Component	Single reaction volume
	Mix (2×)	10.00 μL
	Primer (F)	0.20 μL

	Primer (R)	0.20 μL
	ddH_2O	7.60 μL
	DNA Template	2.00 μL
	Total volume	20.00 μL
Droplet Generation	Oil : 70.00 μL	Sample : 20.00 μL
PCR reaction conditions	Step 1	95°C, 5.00, Ramp 2°C/s
	Step 2	95°C, 0:30, Ramp 2°C/s
	Step 3	52°C, 1:00, Ramp 2°C/s
	Step 4	GOTO step 2, 40×
	Step 5	4°C, 5:00, Ramp 2°C/s
	Step 6	90°C, 5:00, Ramp 2°C/s
	Step 7	12℃，∞

Each test was repeated three times.

Table S6. Parameters of the co-occurrence network.

Parameters	push-flow trough composting	membrane-covered composting
Number of total edges	128.00	102.00
Number of total nodes	82.00	88.00
Network diameter	6.00	4.00
Network density	0.04	0.03
Network modularity	0.67	0.67
Average path length	2.47	2.58
Average degree	3.12	2.32
